# Selectively increasing GHS-R1a expression in dCA1 excitatory/inhibitory neurons have opposite effects on memory encoding

**DOI:** 10.1186/s13041-021-00866-8

**Published:** 2021-10-12

**Authors:** Nan Li, Na Li, Fenghua Xu, Ming Yu, Zichen Qiao, Yu Zhou

**Affiliations:** 1 Department of Physiology and Pathophysiology, School of Basic Medical Sciences, Qingdao University, Qingdao, 266071 Shandong China; 2 Institute of Brain Sciences and Related Disorders, Qingdao University, Qingdao, 266071 Shandong China; 3 Department of Rehabilitation Medicine, Affiliated Hospital of Qingdao University, Qingdao, 266000 Shangdong China

**Keywords:** GHS-R1a, Ghrelin, Memory, Hippocampus, Interneuron, Alzheimer’s disease

## Abstract

**Aim:**

Growth hormone secretagogue receptor 1a (GHS-R1a) is widely distributed in brain including the hippocampus. Studies have demonstrated the critical role of hippocampal ghrelin/GHS-R1a signaling in synaptic physiology, memory and cognitive dysfunction associated with Alzheimer’s disease (AD). However, current reports are inconsistent, and the mechanism underlying memory modulation of GHS-R1a signaling is uncertain. In this study, we aim to investigate the direct impact of selective increase of GHS-R1a expression in dCA1 excitatory/inhibitory neurons on learning and memory.

**Methods:**

Endogenous GHS-R1a distribution in dCA1 excitatory/inhibitory neurons was assessed by fluorescence in situ hybridization. Cre-dependent GHS-R1a overexpression in excitatory or inhibitory neurons was done by stereotaxic injection of *aav-hSyn-DIO-hGhsr1a-2A-eGFP* virus in dCA1 region of vGlut1-Cre or Dlx5/6-Cre mice respectively. Virus-mediated GHS-R1a upregulation in dCA1 neurons was confirmed by quantitative RT-PCR. Different behavioral paradigms were used to evaluate long-term memory performance.

**Results:**

GHS-R1a is distributed both in dCA1 excitatory pyramidal neurons (αCaMKII^+^) and in inhibitory interneurons (GAD67^+^). Selective increase of GHS-R1a expression in dCA1 pyramidal neurons impaired spatial memory and object-place recognition memory. In contrast, selective increase of GHS-R1a expression in dCA1 interneurons enhanced long-term memory performance. Our findings reveal, for the first time, a neuronal type-specific role that hippocampal GHS-R1a signaling plays in regulating memory. Therefore, manipulating GHS-R1a expression/activity in different subpopulation of neurons may help to clarify current contradictory findings and to elucidate mechanism of memory control by ghrelin/GHS-R1a signaling, under both physiological and pathological conditions such as AD.

**Supplementary Information:**

The online version contains supplementary material available at 10.1186/s13041-021-00866-8.

Ghrelin is the only identified orexigenic gastric hormone that promotes feeding, and is critical for metabolism regulation in both human and rodents [1]. It has been reported that only acylated ghrelin (AG) in circulation is capable of binding to ghrelin receptor, the growth hormone secretagogue receptor 1a (GHS-R1a), which is widely distributed in multiple brain regions including the hippocampus [2]. In contrast, unacylated ghrelin (UAG), the most abundant form of circulating ghrelin, is unable to activate GHS-R1a [3, 4].

Studies have highlighted intriguing contradictory roles that ghrelin and GHS-R1a play in regulating multiple neuronal functions such as learning and memory, other than nutrient sensing and metabolic control [5]. For instance, pharmacological studies have reported that ghrelin activating GHS-R1a either facilitates or impairs memory processes [6, 7]. Genetic GHS-R1a null mutation also gave rise to opposite effects on hippocampus-dependent memory encoding [8, 9]. To date, the reason for those conflicting findings remains unclear, and the mechanism underlying memory modulation by GHS-R1a signaling is not well explored.

It is important to note that GHS-R1a displays two uncommon features that may greatly contribute to its functional complexity, extremely high constitutive activity [10] and multiple downstream signaling pathways involved under different experimental conditions [11]. In particular, recent studies have illustrated physiological importance of constitutive activity of GHS-R1a in regulating food intake, growth hormone release, and memory processes [12, 13]. Therefore, altered GHS-R1a expression might lead to distinct biological outcomes from that of ghrelin-dependent activation, under both physiological and pathological conditions like AD. Therefore, in this study, we sought to investigate the direct effect of increasing GHS-R1a expression in specific populations of dCA1 neurons on hippocampus-dependent learning and memory.

Endogenous GHS-R1a distribution in both excitatory and inhibitory dCA1 neurons was confirmed by fluorescent in situ hybridization assays (Fig. 1a). Cre-dependent GHS-R1a-expressing virus (*aav-hSyn-DIO-hGhsr1a-2A-eGFP*) or control virus (*aav-hSyn-DIO-eGFP*) was delivered in dCA1 of Vglut1-Cre or Dlx5/6-Cre male mice (3–4 month old) respectively to selectively increase GHS-R1a expression in excitatory or inhibitory neurons in dorsal hippocampus. GFP fluorescence in dCA1 region indicated successful viral transfection and virus-mediated GHS-R1a expression in pyramidal neurons or interneurons 3 weeks after injection (Fig. 1b, i). Virus-mediated GHS-R1a expression in dorsal hippocampus was quantified by RT-qPCR analyses (Fig. 1c, j). The detailed methods were described in Additional file 1.

**Fig. 1:**
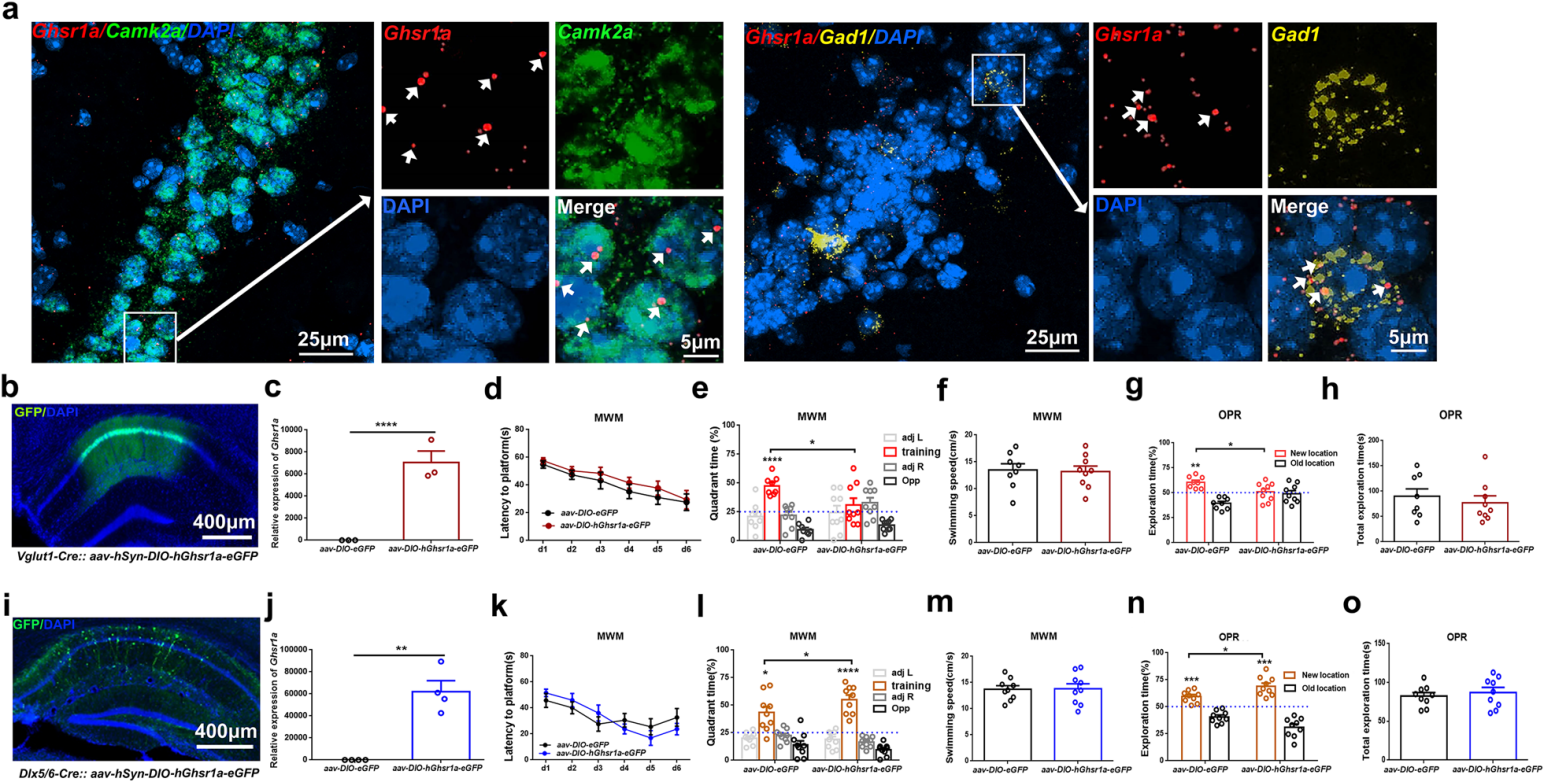
Selective GHS-R1a upregulation in dCA1 pyramidal neurons or interneurons has opposite effect on hippocampus-dependent memory encoding. **a** Representative fluorescent in situ hybridization images showing endogenous *Ghsr1a* expression in both excitatory and inhibitory dCA1 neurons of C57BL/6J mice. *Ghsr1a* (red), *Camk2a* (green), *Gad1* (yellow), DAPI (blue). Arrowheads (white) indicate *Ghsr1a* signals within *Camk2a-* or *Gad1*-expressing neurons. **b, i** Representative fluorescent images of dorsal hippocampus taken 4 weeks after virus injection. Vglut1-Cre mice (**b**), Dlx5/6-Cre mice (**i**). GFP (green), DAPI (blue). **c, j** RT-qPCR analyses showing increased *hGhsr1a* expression in dorsal hippocampus 4 weeks after delivery of *hGhsr1a-expressing* virus. Vglut1-Cre mice (**c**), n = 3 per group; Dlx5/6-Cre mice (**j**), n = 4 per group. **d**–**h, k**–**o** Learning and memory performance. Vglut1-Cre mice (**d**–**h**), Dlx5/6-Cre mice (k–o). **d**–**f, k**–**m** Morris water maze assays. **d, k** GHS-R1a upregulation does not affect spatial learning. **e, l **Spatial memory tested 24 h after the 6th day training. Elevated GHS-R1a in excitatory neurons impairs spatial memory (**e**), while increased GHS-R1a expression in inhibitory neurons enhances spatial memory (**l**). **f, m** Averaged swimming speed during probe test. **g, h, n–o** Object-place recognition (OPR) assays. **g** Cre-dependent GHS-R1a upregulation in excitatory neurons impairs OPR memory. **n** Cre-dependent GHS-R1a upregulation in inhibitory neurons improves OPR memory. **h, o** Total object exploration time during OPR test. Vglut1-Cre mice with GHS-R1a-expressing virus (n = 9), Vglut1-Cre mice with control virus (n = 8), Dlx5/6-Cre mice, n = 9 per group. All data is shown as means ± SEM. Two-way repeated-measure ANOVA with Sidak’s multiple comparisons test for (**d**, **e**, **g**, **k**, **l**, **n**), unpaired *t* test for (**c**, **f**, **h**, **j**, **m**, **o**), ****P < 0.0001, ***P < 0.001, ** *P* < 0.01 or *P < 0.05 means significant difference, n.s. means no significance

The effect of increasing GHS-R1a expression on hippocampus-dependent learning and memory performance was evaluated 4 weeks after viral injection. We found that selective GHS-R1a upregulation in dCA1 excitatory pyramidal neurons impairs hippocampus-dependent memory processes. Specifically, Vglut1-Cre mice transfected with *aav-hSyn-DIO-hGhsr1a-2A-eGFP* virus exhibited poor spatial memory (Fig. 1d, f), impaired object-place recognition (OPR) memory (Fig. 1g, h), in comparison to Vglut1-Cre mice receiving control *aav-hSyn-DIO-eGFP* virus injection. In contrast, we found that Dlx5/6-Cre mice receiving *aav-hSyn-DIO-hGhsr1a-2A-eGFP* virus displayed better spatial memory (Fig. 1k, m) and OPR memory (Fig. 1n, o) than control Dlx5/6-Cre mice, indicating that selective GHS-R1a upregulation in dCA1 inhibitory interneurons improves hippocampus-dependent memory. Our findings thus reveal, for the first time, that elevated GHS-R1a expression selectively in dCA1 excitatory/inhibitory neurons differentially regulates memory encoding. It will be interesting to know what kind of GHS-R1a activity, ligand-dependent or ligand-independent or both, mediates the differential effect of elevated GHS-R1a on memory. Additional studies are also needed to explore synaptic mechanisms and signaling cascades mediating these cell-type specific effects of GHS-R1a activation on memory.

The hippocampus is a complex network tightly regulated by interactions between excitatory pyramidal neurons and inhibitory interneurons. Although represent a minority in the hippocampus, interneurons play a critical role in shaping network activities [14]. However, no previous study has reported the physiological importance of ghrelin/GHS-R1a signaling in hippocampal interneurons. In this study, we uncovered its memory improvement effect by directly increasing GHS-R1a expression in dCA1 inhibitory neurons, as opposed to the memory impairment effect of GHS-R1a upregulation in excitatory neurons. Our current findings, together with on-going study based on conditional GHS-R1a knockout mice, will help to reveal causal association between hippocampal GHS-R1a expression and memory. In addition, accumulating evidence suggests a correlation between altered GHS-R1a expression and AD pathogenesis [15]. Therefore, it is necessary to test the direct impact of manipulating hippocampal GHS-R1a expression on AD memory impairment.

In conclusion, our findings reveal, for the first time, that elevated GHS-R1a expression selectively in dCA1 excitatory/inhibitory neurons differentially regulates memory encoding. It also suggests a causal relationship between hippocampal GHS-R1a expression and memory.

## Supplementary Information

**Additional file 1.** Selectively increasing GHS-R1a expression in dCA1 excitatory/inhibitory neurons have opposite effects on memory encoding.

## Data Availability

The detailed methods were described in Additional file 1.

## Abbreviations

GHS-R1a: Growth hormone secretagogue receptor 1a
*hGhsr1a*: Human *Ghsr1a*
AG: Acylated ghrelin
UAG: Unacylated ghrelin
Aav: Adeno-associated virus
GFP: Green fluorescent protein
PCR: Polymerase chain reaction
OPR: Object-place recognition
αCaMKII: Ca^2^^+^/calmodulin-dependent protein kinase IIα
AD: Alzheimer’s disease
FISH: Fluorescence in situ hybridization
hSyn: Human synapsin I
Dlx5/6: Distal-less homeobox 5/6
Vglut1: Vesicular glutamate transporter 1
*Gad1*: Glutamate decarboxylase 1 (GAD1)
