# Supplementary material for: Selectively increasing GHS-R1a expression in dCA1 excitatory/inhibitory neurons have opposite effects on memory encoding
Source: Mol Brain. 2021 Oct 12;14:157. doi: 10.1186/s13041-021-00866-8 (PMC8513281; doi:10.1186/s13041-021-00866-8)
Supplement: Supplementary file 1 — Additional file 1. Selectively increasing GHS-R1a expression in dCA1 excitatory/inhibitory neurons have opposite effects on memory encoding. [file 13041_2021_866_MOESM1_ESM.docx]

**Materials and methods**

1. ***Mice***

Vglut1-Cre mice (B6;129S-Slc17a7^tm1.1(cre)Hze^/J, Jax 023527) and Dlx5/6-Cre mice (Tg(dlx5a-cre)1Mekk/J, Jax 008199) were purchased from JAX, and cross with C57BL/6J mice. C57BL/6J mice were purchased from the Vital River Laboratory Animal Technology Co. (Beijing, China). Genotypes were confirmed with PCR analyses. Mice were group-housed on a 12 h: 12 h light/dark cycle, at 20~ 22-℃ room temperature with free access to food and water. Only adult male littermate mice at age of 12-20 weeks old were used for behavioral experiments. The Chancellor’s Animal Research Committee at Qingdao University approved all animal protocols in this study, in accordance with National Institutes of Health guidelines.

1. ***Virus injection in dCA1 region of the hippocampus***

Mice were anaesthetized with isoflurane and head-fixed on a stereotaxic frame. High titer AAV virus (>10^13 GC/ml) were bilaterally introduced into dCA1 region through a glass needle connected to a WPI Nanoliter 2000 at a flow rate of 0.03 µl/min. GHS-R1a-expressing virus (*aav-hSyn-DIO-hGhsr1a-2A-eGFP,*) and control virus (*aav-hSyn-DIO-eGFP*) were prepared by Shanghai Obio Technology (China). The four dCA1 injection coordinates were AP −1.8 mm, ML ± 1 mm, DV −1.5 mm and AP −2.5 mm, ML ± 2 mm, DV −1.6 mm relative to bregma. After virus (0.15 µl) was delivered to each point, the glass needle was left in original position for an additional 10 min to ensure optimal diffusion. Viral transfection in dCA1 was confirmed by GFP fluorescence under an Olympus VS120 virtual slide microscope. Virus-mediated GHS-R1a expression in dorsal hippocampus was measured by RT-qPCR analyses. Experiments were performed 4 weeks after virus delivery.

***3. Behavioral training and testing***

All behavioral experiments were done with dim light and during light cycle. Mice were habituated in experimental room for at least 2 h before starting experiments. Behaviors were video-tracked and analyzed with Noldus EthoVision XT software.

*Morris water maze (MWM)*. MWM assays were conducted in a circular water pool (120 cm in diameter, 30 cm in depth) that was divided into four even quadrants. During training, an invisible escape platform (10 cm in diameter) was kept 0.5 cm underneath water surface. Mice were trained with 4 trails/2 blocks/day for 6 days, with an inter-block interval of 1 h and an inter-trial interval of 30 s. A training trial ended whenever mice climbed onto the hidden platform or a cut-off time of 60 s reached, whichever came earlier. A probe test was carried out to evaluate spatial memory at the following time points: 1h after the 3^rd^ day, the 5^th^ day training, and 24h after the 6^th^ day training. During testing, the hidden platform was removed from the pool and mice were allowed to navigate for 60 s.

*Object-place recognition (OPR)*. OPR training and testing were carried out in a non-transparent chamber (27.3 × 27.3 × 20.3 cm) with visual cures on the wall. During training, mice freely explored two identical objects inside the chamber for 10 min. OPR memory was tested 24 hrs after training with one object transferred to a new location. Percentage of time mice exploring object in new or old place was measured during a 5 min testing. Recognition Memory Index (%) was calculated as (exploration time for object in new place/total objects exploration time) x 100%.

***4. Histology and immunostaining***

Mice were perfused first with 0.9% saline then with 4% paraformaldehyde (PFA). Extracted whole brain were post-fixed in 4% PFA for additional 4~6 h, and then dehydrated in 30% sucrose for 48 hrs. Frozen brains were sectioned into 40 μm coronal slices with a Leica cryostat. The location of cannulas were checked with methylene blue staining. Only mice with proper cannula placement in the dCA1 were included in further analyses. Rabbit anti-GFP primary antibody (Abcam, 1:1000) and Alexa-488-conjugated goat anti-rabbit secondary antibody (Abcam, 1:500) were used for GFP staining. Slices were counterstained with 4′,6-diamidino-2-Phenylindole (DAPI). Images were scan collected with virtual slide microscope (VS120, Olympus) with 10x or 20x objective lens.

***5. Fluorescence in situ hybridization (FISH)***

FISH was performed with RNAscope Multiplex Fluorescent Reagent Kit V2 (ACD, 323100) following the manufacturer’s instruction. Briefly, fresh brains were instantly frozen in isopentane, and coronal slices (14 μm in thickness) were mounted on SuperFrost Plus Gold slides (Fisher Scientific). After fixation in 4% PFA for 15 min at 4 ˚C, brain slices were dehydrated in 50%, 70%, and 100% ethanol in sequence, and air-dried at room temperature. RNA probes for *Camk2a*, *Gad1* and *Ghsr1a* were purchased from ACD. TSA Plus fluorescein, TSA Plus Cyanine 3 and TSA Plus Cyanine 5 were used to develop fluorescent signals that can be visualized and captured by a Leica LAS-X confocal microscope with a 63x oil-immersion objective lens. Gain, threshold, and black levels remained unchanged during individual experiment.

***6. Quantitative reverse transcription PCR (RT-qPCR)***

Total RNA was extracted from dorsal hippocampus with the PureLink^TM^ RNA Mini Kit (Thermo Fisher Scientific). RNA quantity and quality were measured using a NanoDrop 2000 Spectrophotometer (Thermo Fisher Scientific). Complementary DNA was synthesized from 1µg of total RNA with SuperScript^TM^ III Reverse Transcriptase (Thermo Fisher Scientific). PCR-based quantification of *hGhsr1a* was done using a MasterCycler® ep realplex PCR system (Eppendorf) and a QuantiFast SYBR Green PCR Kit (Qiagen). The PCR cycling parameters were as follows: 95 ℃ for 5 min, followed by 40 cycles of PCR reaction at 95 ℃ for 5 s, 60 ℃ for 30 s, 72 ℃ for 30 s. 2^-ΔΔCT^ method was used to normalize CT values against housekeeping gene *Actb* and quantify virus-mediated GHS-R1a expression. PCR primer sequences (Thermo Fisher Scientific) used were as follows: *Ghsr-F GAAAATGCTGGCTGTAGTGGTG, Ghsr-R GACAAAGGACACGAGGTTGC; Actb-F CATCCGTAAAGACCTCTATGCCAAC, Actb-R ATGGAGCCACCGATCCACA.* Triplicates were done for each sample.

***7. Statistical analysis***

Results were expressed as means ± SEMs. Data were analyzed using unpaired *t* test, one-sample *t* test, or two-way ANOVA with appropriate multiple comparisons test. *P < 0.05* indicates significant difference between groups. Statistical analysis was performed with GraphPad Prism 6.0 (GraphPad Software).
